# Influence of cognitive reserve on risk of depression and subsequent dementia: A large community-based longitudinal study

**DOI:** 10.1192/j.eurpsy.2024.1762

**Published:** 2024-06-04

**Authors:** Wenzhe Yang, Jiao Wang, Abigail Dove, Yonghua Yang, Xiuying Qi, Marc Guitart-Masip, Goran Papenberg, Weili Xu

**Affiliations:** 1 School of Public Health, Tianjin Medical University, Tianjin, China; 2 Tianjin Key Laboratory of Environment, Nutrition and Public Health, Tianjin, China; 3 Department of Epidemiology, College of Preventive Medicine, Army Medical University (Third Military Medical University), Chongqing, China; 4 Aging Research Center, Department of Neurobiology, Care Sciences and Society, Karolinska Institutet, Stockholm, Sweden; 5 Department of Rehabilitation Medicine, Xiaogan Hospital of Traditional Chinese Medicine, Xiaogan, China; 6 Center for Psychiatry Research, Region Stockholm, Stockholm, Sweden; 7 Center for Cognitive and Computational Neuropsychiatry (CCNP), Karolinska Institutet, Stockholm, Sweden

**Keywords:** cognitive reserve, dementia, depression, multi-state model, UK Biobank

## Abstract

**Background:**

Cognitive reserve (CR) has been linked to dementia, yet its influence on the risk of depression and related outcomes remains unknown. We aimed to examine the association of CR with depression and subsequent dementia or death, and to assess the extent to which CR is related to depression-free survival.

**Methods:**

Within the UK Biobank, 436,232 participants free of depression and dementia were followed. A comprehensive CR indicator (low, moderate, and high) was created using latent class analysis based on information on education, occupation, mentally passive sedentary behavior, social connection, confiding with others, and leisure activities. Depression, dementia, and survival status were ascertained through self-reported medical history and/or linkages to medical records. Data were analyzed using multi-state Markov model and Laplace regression.

**Results:**

Over a median follow-up of 12.96 years, 16,560 individuals developed depression (including 617 with subsequent dementia) and 28,655 died. In multivariable multi-state models, compared with low CR, high CR was associated with lower risk of depression (hazard ratio 0.53 [95% confidence interval 0.51–0.56]) and lower risk of post-depression dementia (0.55 [0.34–0.88]) or death (0.69 [0.55–0.88]) in middle-aged adults (aged <60 years). In Laplace regression, the depression-free survival time was prolonged by 2.77 (2.58–2.96) years in participants with high compared to low CR.

**Conclusions:**

High CR is associated with lower risks of depression and subsequent transitions to dementia and death, particularly in middle age. High CR may prolong depression-free survival. Our findings highlight the importance of enhancing CR in the prevention and prognosis of depression.

## Introduction

Depression is a common mental disorder that affects 350 million people, equivalent to 5% of the adult population worldwide [1]. It is estimated that depression ranks first in terms of the global burden of mental health-related disease [2]. Depression is one of the leading causes of avoidable disability, which brings great suffering to individuals and families, impairs social functioning, and is related to physical illnesses and suicide [1]. As underscored by the World Psychiatric Association Commission, prevention and intervention are essential to alleviating the burden of depression [1]. Focusing on modifiable risk factors and promoting primary prevention of depression constitute a public health priority.

Compared with the general population, people suffering from depression may be more susceptible to dementia [3, 4]. Accumulating evidence indicates that depression and dementia share common neurobiological processes, suggesting shared risk factors for the two neuropsychiatric disorders [3, 5]. Cognitive reserve (CR), developed through lifetime cognitively stimulating or demanding experiences, has been proposed as an important modifiable factor in reducing dementia risk [6, 7], and it is plausible that enhancing CR might also help buffer depression risk and prevent subsequent dementia. On the contrary, depression has been linked to elevated mortality risk and shortened life expectancy in many studies, including our previous work [8–10]. Therefore, tertiary prevention is also needed to stave off the development of disease to a worse outcome for those with depression.

Previous studies have mostly focused on the stage before the onset of depression, showing lower risks of incident depression with individual CR-related factors, such as higher education, less engagement in mentally passive sedentary behaviors, and greater social participation [11–13]. In addition, our previous research using data from the Swedish Twin Registry has suggested that higher education might attenuate dementia risk related to mid-life depression [4]. To the best of our knowledge, however, no literature to date has investigated and compared the influence of a combined CR indicator on both the onset of depression and the subsequent transition to dementia or death. Importantly, a holistic understanding of how modifiable risk factors play a role in different stages of disease progression contributes to optimizing strategies for multi-level prevention.

In this study, we aimed to (1) examine the association of a composite CR indicator with the risk of depression and subsequent transition to dementia and death and (2) estimate the extent to which CR might prolong depression-free survival using data from the UK Biobank.

## Methods

### Study population

Data used in this study were derived from the UK Biobank, a large population-based longitudinal study. Between 2006 and 2010, more than 500,000 individuals aged 37–73 years were recruited and underwent comprehensive assessments at 22 assessment centers across the United Kingdom. All enrolled participants provided informed and written consent. Of 502,412 participants in the baseline examination, we excluded 65,716 with a history of depression (*n =* 65,543) or dementia (*n =* 238) at recruitment, 268 who developed depression after the occurrence of dementia, and 196 who developed both dementia and depression on the same date. Overall, 436,232 participants were included in the current study (Supplementary Figure S1).

The UK Biobank received ethical approval from the North West Multi-Centre Research Ethics Committee (21/NW/0157), and our work was performed under the UK Biobank application number 67048 (PI: Weili Xu).

### Data collection

At baseline, information on participants’ age, sex, race (white versus mixed, Asian or Asian British, black or black British, Chinese, or other ethnic groups), smoking status (never, previous, or current), alcohol consumption (never, previous, or current), and physical activity was self-reported through computerized touch-screen questionnaires. Physical activity was measured as total metabolic equivalents (MET) per week using the modified version of the International Physical Activity Questionnaire and classified as low (<600 MET-min/week), moderate (600 to <3000 MET-min/week), or high (≥3000 MET-min/week) [14]. Body weight and height were measured, with body mass index (BMI) calculated as weight (kg)/(height (m)^2^). Hypertension was identified based on systolic blood pressure ≥ 140 mm Hg, diastolic blood pressure ≥ 90 mm Hg, self-reported history of hypertension, use of antihypertensive drugs, or medical records. Diabetes was defined as the presence of hemoglobin A1c ≥6.5%, fasting plasma glucose ≥126 mg/dL, self-reported history of diabetes, use of glucose-lowering medications, or medical records. Heart disease (including myocardial infarction, angina, atrial fibrillation, and heart failure) and stroke were ascertained through medical records and self-reported medical history.

### Assessment of CR and generation of CR indicator

CR was assessed based on six factors including educational level, occupational complexity, mentally passive sedentary behavior, social connection, confiding in others, and leisure activity engagement, as defined in previous studies [15–18]. All information about these factors was self-reported at baseline.

Educational level was determined according to the years of regular schooling converted based on the International Standard Classification of Education scale, divided into (1) no educational qualifications (equal to 7 years), (2) Certificate of Secondary Education, Ordinary levels/General Certificate of Secondary Education (equal to 10 years), Advanced levels/Advanced Subsidiary levels or equivalent (equal to 13 years), (3) other professional qualifications (equal to 15 years), (4) National Vocational Qualification, Higher National Diploma, Higher National Certificate or equivalent (equal to 19 years), or (5) college/university degree (equal to 20 years) [19].

Occupational complexity was assessed based on participants’ current (or, for retired people, longest-held) occupation and categorized according to the UK Standard Occupational Classification 2000 system, which was developed by the UK Office of National Statistics [20]. Occupation was further classified into one of the eight socioeconomic categories in the National Statistics Socio-economic Classification (SEC) [21], coded as ordinal variables ranging from 1 to 8, where lower values indicate higher occupational complexity and attainment (i.e., jobs requiring more thought and higher skill levels) [22]. Occupational complexity was categorized into five levels: (1) never worked and long-term unemployed (SEC 8) or routine occupations (SEC 7), (2) semi-routine occupations, small employers and own account workers, or lower supervisory and technical occupations (SEC 6–4), (3) intermediate occupations (SEC 3), (4) lower managerial and professional occupations (SEC 2), and (5) higher managerial and professional occupations (SEC 1).

Mentally passive sedentary behavior was assessed based on the time (in hours/day) that participants spent in watching television, categorized as (1) ≥4, (2) 3–3.9, (3) 2–2.9, or (4) <2.

Social connection was measured based on the frequency of participants visiting or being visited by friends or family, divided into (1) no friends/family outside household or about once a month or less, (2) about once a week, (3) 2–4 times a week, or (4) almost daily.

Confiding in others was determined based on the frequency of participants confiding in someone close to them, classified as (1) never or almost never, (2) about once a month or less, (3) 1–4 times a week, or (4) almost daily.

Leisure activity engagement was assessed according to the number of leisure activities (including sports club or gym, pub or social club, religious group, adult education class, and other group activity) participants engaged in at least once a week, classified as three levels: (1) low (none), (2) moderate (1 activity), or (3) high (2–5 activities).

A composite CR indicator was constructed using latent class analysis (LCA) based on these six factors. LCA is a well-validated statistical approach that can identify hidden clusters by grouping multiple observed categorical variables (i.e., CR-related factors) into a latent variable (i.e., the CR indicator) with mutually exclusive latent classes. Three latent classes were identified after comprehensively considering statistics regarding model selection (with a relatively lower Bayesian information criterion value) and the uncertainty of posterior classification (with mean posterior probabilities in all latent classes >0.70), and they respectively represented a high (characterized by higher levels of education, occupational complexity, confiding in others, and leisure activity engagement as well as less mentally passive sedentary behavior), moderate (characterized by moderate levels of all CR-related factors), and low level (characterized by a higher level of social connection but less favorable levels of other CR-related factors) of CR according to the item-response probabilities (Supplementary Table S1). Similar calculations have been described previously [23, 24], and the methodology details are available in Supplementary Material S1.

### Ascertainment of depression, dementia, and death

Incident depression was identified through hospital admissions data (i.e., Hospital Episode Statistics-Admitted Patient Care in England, Scottish Morbidity Records-General/Acute Inpatient and Day Case Admissions in Scotland, and Patient Episode Database in Wales), primary care records, self-reported diagnoses of depression, and death registries. These events were recorded and coded based on the International Classification of Diseases, Version 10, and codes F32–F33 were used. Prevalent depression was detected at baseline using the hospital admissions data, primary care records, self-reported diagnoses, and the Patient Health Questionnaire-2 (PHQ-2) which assessed the frequency of depressed mood and anhedonia over the past 2 weeks, with cutoff ≥2 reflecting possible depression [25].

Dementia was ascertained based on algorithmic definitions developed by the UK Biobank outcome adjudication group, which combined multiple data sources including hospital admissions, self-reported diagnoses of dementia/Alzheimer’s disease/cognitive impairment, and/or death registries. Post-depression dementia was defined as having dementia after the occurrence of depression. The earliest recorded date of occurrences of depression and dementia were used and compared to ensure the chronological order of events. Data on deaths from all causes were extracted via linkage to national death registries.

### Statistical analysis

Baseline characteristics of participants according to CR level were compared using a one-way analysis of variance for normally distributed continuous variables or chi-square test for categorical variables.

**Figure 1.**
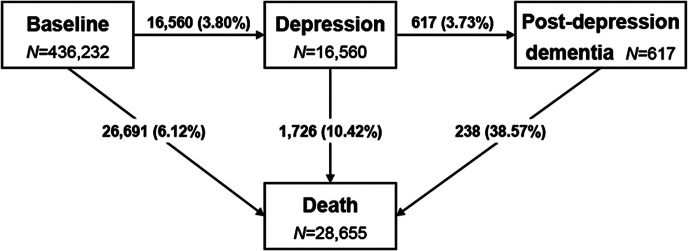
Schematic representation of multistate model. State-specific numbers of participants were reported in boxes, and numbers (percentages) of participants in transitions from baseline to depression, subsequently to dementia, and ultimately to death were reported on arrows.

A multi-state Markov model was used to assess the influence of CR (reference group: low CR) on the risk of incident depression and subsequent dementia and/or death. Results are presented as transition-specific hazard ratios (HRs) and 95% confidence intervals (CIs) of depression and related outcomes. The proportional hazards assumption was checked using Schoenfeld residuals and no violations were detected. Follow-up time was calculated as the time from baseline to death or end of follow-up (January 31, 2022), whichever came first. In this study, five transition phases were considered: (1) baseline to depression, (2) depression to dementia, (3) baseline to death without depression, (4) depression to death without dementia, and (5) post-depression dementia to death (Figure 1). For participants whose depression/dementia diagnosis and death were recorded on the same date, the entry date of theoretically prior state was calculated as the entry date of the latter state minus the median interval time of corresponding stage (577 days for transition 4, *n =* 13; 304 days for transition 5, *n =* 21) [26]. We only considered the first entry into a state, and no reversal of state was allowed.

To further explore the role of CR in the prevention of depression, we assessed the probability and duration of depression-free survival (i.e., an initial state without any transition to depression, post-depression dementia, or death) according to CR level. A combined outcome was defined as either incident depression or death. Cox proportional hazard regression was used to examine the longitudinal association between CR and the combined outcome. Follow-up time was calculated as the time from baseline until the earliest occurrence of depression, death, or end of follow-up. Laplace regression was used to estimate the absolute percentile difference in time until the occurrence of outcome according to CR level, so the results indicated depression-free survival times. Because nearly 10% of participants developed depression or died, we modeled and predicted differences in time (in years) by which the first 10% of participants would experience the outcome.

Given possible differences in prevalence and etiology of depression and dementia, as well as the complex relationship between the two diseases, at different ages, we also analyzed the aforementioned associations after stratifying by age group (middle age [<60 years, *n =* 244,368] versus older age [≥60 years, *n =* 191,864]). A multiplicative interaction was tested by incorporating the two factors (i.e., the CR indicator and age group) and their cross-product term in the same models. All analyses were adjusted for age, sex, race, smoking status, alcohol consumption, physical activity, BMI, hypertension, diabetes, heart disease, and stroke. Missing data were imputed using the fully conditional specification, with estimates pooled across five iterations.

Several supplementary analyses for the multi-state analysis were performed: (1) to minimize the influence of reverse causation, we excluded the cases of depression (*n =* 943) or dementia (*n =* 30) that occurred during the first year of follow-up, (2) we recalculated the entering date of the prior state using a 0.5 day of time interval for participants who entered different states on the same date in transitions 4 and 5 [27], and (3) we further adjusted for the Townsend deprivation index, a variable reflecting neighborhood-level socioeconomic status. Statistical analyses were performed using Stata SE 15.0 (StataCorp, College Station, TX, USA) and R software (version 4.3.0). Results with a 2-sided *P* value <0.05 were considered statistically significant.

## Results

### Characteristics of the study population

At baseline, among 436,232 participants (mean age 56.64 ± 8.11 years, 52.80% women, 90.83% white), 156,337 (35.84%) were of high CR, 194,912 (44.68%) of moderate CR, and 84,983 (19.48%) of low CR. Baseline characteristics of participants by CR level are shown in Table 1. Compared with participants with low CR, those with moderate or high CR were younger, more likely to be non-smokers, current drinkers, and to have lower BMI, a lower level of physical activity, and a lower prevalence of hypertension, diabetes, heart disease, or stroke.

**Table 1.** Baseline characteristics of the study population by different levels of cognitive reserve

Characteristics	Cognitive reserve			*P*
	Low (*n =* 84983)	Moderate (*n =* 194912)	High (*n =* 156337)	
Age (years)	60.23 ± 7.01	56.23 ± 8.13	55.20 ± 8.08	<0.001
Sex				<0.001
Women	45006 (52.96)	105717 (54.24)	79608 (50.92)	
Men	39977 (47.04)	89195 (45.76)	76729 (49.08)	
White race	78609 (92.50)	181080 (92.90)	136558 (87.35)	<0.001
Smoking status				<0.001
Never	38717 (45.56)	107354 (55.08)	96391 (61.66)	
Previous	33130 (38.98)	68015 (34.90)	49030 (31.36)	
Current	13136 (15.46)	19543 (10.03)	10916 (6.98)	
Alcohol consumption				<0.001
Never	6083 (7.16)	7655 (3.93)	5706 (3.65)	
Previous	4750 (5.59)	5360 (2.75)	3910 (2.50)	
Current	74150 (87.25)	181897 (93.32)	146721 (93.85)	
Physical activity				<0.001
Low	13388 (15.75)	30742 (15.77)	26235 (16.78)	
Moderate	34916 (41.09)	90750 (46.56)	84746 (54.21)	
High	36679 (43.16)	73420 (37.67)	45356 (29.02)	
Body mass index (kg/m^2^)	28.47 ± 5.01	27.54 ± 4.68	26.48 ± 4.38	<0.001
Hypertension	34828 (40.98)	56940 (29.21)	36718 (23.49)	<0.001
Diabetes	7521 (8.85)	9457 (4.85)	5778 (3.70)	<0.001
Heart disease	8879 (10.45)	9385 (4.81)	5258 (3.36)	<0.001
Stroke	2414 (2.84)	2416 (1.24)	1374 (0.88)	<0.001

*Note:* Data are presented as mean ± standard deviation or number (%).

### Association of CR with incident depression and its subsequent transition to dementia and death

Over a median follow-up period of 12.96 years (interquartile range: 12.21 to 13.64 years), 16,560 individuals experienced incident depression, of whom 617 subsequently developed dementia. A total of 28,655 deaths from all causes were identified. Among those, 1,726 died after experiencing depression and 238 died after post-depression dementia (Figure 1). Compared with participants with low CR, a lower proportion of those with moderate or high CR experienced each transition, except the transition from post-depression dementia to death (transition 5). The numbers and percentages of events in each transition phase by CR level are shown in Supplementary Table S2.

In multi-state models, compared with participants with low CR, those with high CR had a lower risk of transitioning from baseline to depression (HR 0.53, 95% CI: 0.51–0.56), from depression to dementia (HR 0.79, 95% CI: 0.62–0.98), and from depression to death (0.82, 95% CI: 0.73–0.92). High CR was also associated with a lower risk of mortality from baseline (HR = 0.78, 95% CI: 0.73–0.82), but not from post-depression dementia (HR = 0.97, 95% CI: 0.75–1.26). Furthermore, a similar pattern of associations was observed in middle-aged participants. While among older participants, individuals with high CR had lower risks of depression (HR = 0.64, 95% CI: 0.58–0.70) and the transition from baseline to death (HR = 0.70, 95% CI: 0.64–0.77) than those with low CR. There were significant interactions between high (versus low) CR and age group on all transitions except the transition from post-depression dementia to death (Table 2).

**Table 2.** Hazard ratios (HRs) and 95% confidence intervals (CIs) for associations between cognitive reserve and transitions from baseline to depression, post-depression dementia, and death

Cognitive reserve	HR (95% CI)				
	Baseline to depression	Depression to dementia	Baseline to death	Depression to death	Post-depression dementia to death
All participants					
Moderate versus Low	**0.66 (0.64–0.69)**	0.85 (0.65–1.13)	**0.81 (0.77–0.85)**	**0.89 (0.81–0.97)**	0.85 (0.69–1.06)
High versus Low	**0.53 (0.51–0.56)**	**0.79 (0.62–0.98)**	**0.78 (0.73–0.82)**	**0.82 (0.73–0.92)**	0.97 (0.75–1.26)
Middle age (<60 years)					
Moderate versus Low	**0.57 (0.54–0.61)**	0.68 (0.37–1.25)	**0.52 (0.47–0.57)**	**0.80 (0.67–0.95)**	0.67 (0.37–1.23)
High versus Low	**0.43 (0.40–0.46)**	**0.55 (0.34–0.88)**	**0.49 (0.44–0.54)**	**0.69 (0.55–0.88)**	1.38 (0.68–2.78)
Older age (≥60 years)					
Moderate versus Low	**0.72 (0.67–0.78)**	0.96 (0.71–1.30)	**0.78 (0.72–0.84)**	0.93 (0.83–1.04)	0.89 (0.70–1.12)
High versus Low	**0.64 (0.58–0.70)**	0.84 (0.65–1.09)	**0.70 (0.64–0.77)**	0.93 (0.79–1.09)	1.00 (0.76–1.33)
*P* for interaction between CR (moderate versus low) and age	<0.001	0.038	<0.001	0.098	0.525
*P* for interaction between CR (high versus low) and age	<0.001	0.016	0.001	0.011	0.831

*Note:* Models were adjusted for age, sex, race, smoking status, alcohol consumption, physical activity, body mass index, hypertension, diabetes, heart disease, and stroke, with low cognitive reserve as reference category. HRs (95% CIs) marked in bold indicated significant associations (*P* <  0.05).

### Association of CR with depression-free survival

**Figure 2.**
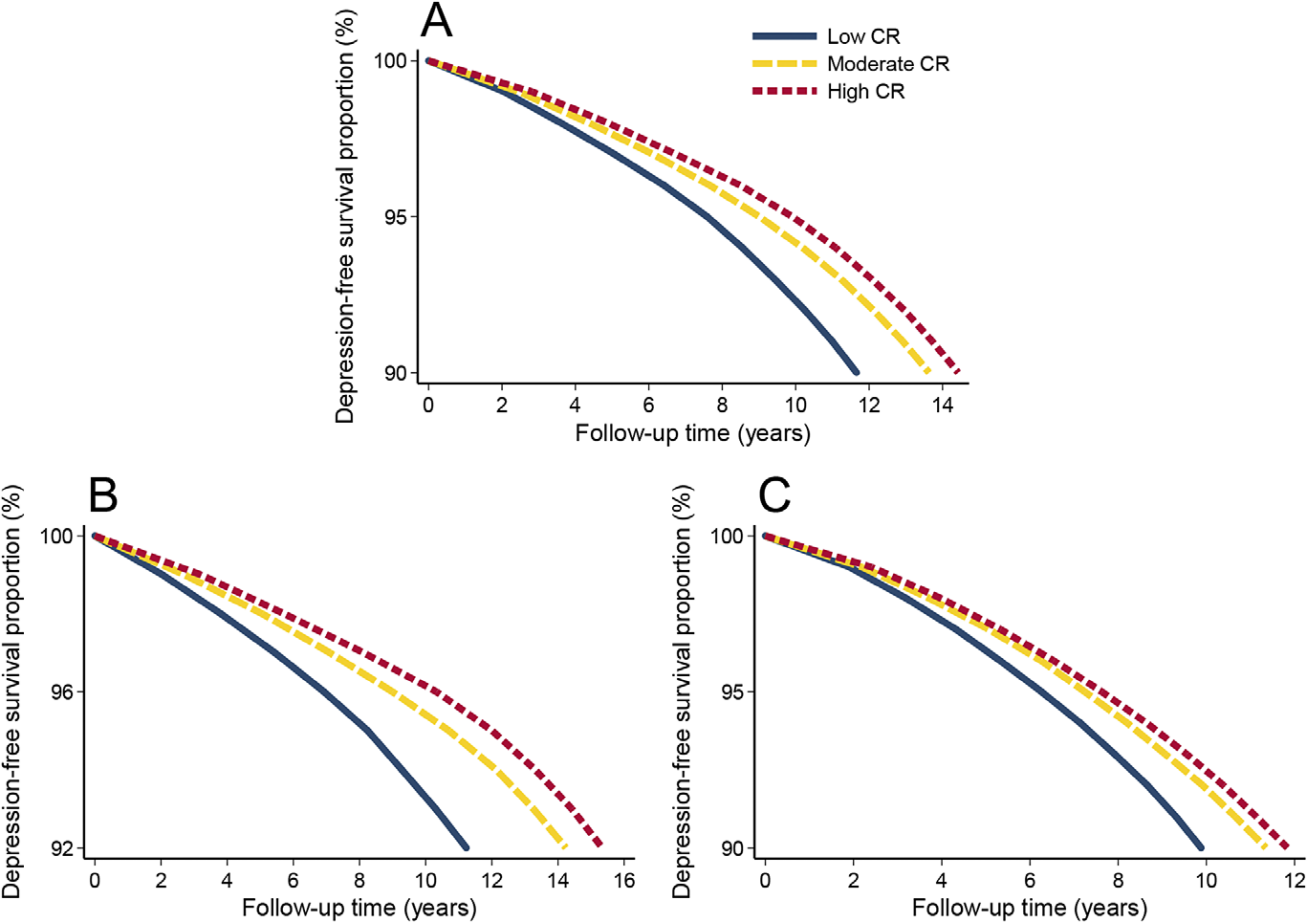
The 10th percentile differences in years of depression-free survival in relation to cognitive reserve (CR) among (A) all participants, (B) participants aged < 60, and (C) participants aged ≥ 60. Models were adjusted for age, sex, race, smoking status, alcohol consumption, physical activity, body mass index, hypertension, diabetes, heart disease, and stroke.

During a median follow-up of 12.91 (interquartile range: 12.13–13.61) years, 43,251 (9.91%) individuals developed depression or died. In Cox models, high CR was associated with a lower risk of depression or death compared with low CR (HR = 0.64, 95% CI: 0.62–0.66). Such associations remained significant across middle and older ages. In Laplace regression, the depression-free survival time was prolonged by 2.77 (95% CI: 2.58–2.96) years among people with high compared with low CR. After age-stratification, the differences in depression-free survival time between individuals with high CR and those with low CR were 4.07 (95% CI: 3.75–4.39) years in middle age and 1.96 (95% CI: 1.71–2.20) years in older age, with significant interactions between CR (for moderate versus low, *P* < 0.001; for high versus low, *P* < 0.001) and age group (Table 3 and Figure 2).

**Table 3.** Hazard ratios (HRs) from Cox models and 10th percentile differences (PDs) in time (years) to incident depression/death from Laplace regression, and 95% confidence intervals (CIs) in relation to cognitive reserve.

Cognitive reserve	No. of participants	Incident depression or death		
		No. of cases	HR (95% CI)	10th PD (95% CI)
All participants				
Low	84983	14076	1.00	0.00
Moderate	194912	17933	0.73 (0.72–0.75)	1.98 (1.81–2.14)
High	156337	11242	0.64 (0.62–0.66)	2.77 (2.58–2.96)
Middle age (<60 years)				
Low	30807	4092	1.00	0.00
Moderate	112965	7638	0.64 (0.61–0.66)	3.01 (2.71–3.31)
High	100596	5236	0.54 (0.52–0.56)	4.07 (3.75–4.39)
Older age (≥60 years)				
Low	54176	9984	1.00	0.00
Moderate	81947	10295	0.78 (0.76–0.80)	1.47 (1.27–1.67)
High	55741	6006	0.70 (0.68–0.73)	1.96 (1.71–2.20)

*Note:* Models were adjusted for age, sex, race, smoking status, alcohol consumption, physical activity, body mass index, hypertension, diabetes, heart disease, and stroke.

### Supplementary analysis

Results were consistent with the original analyses after excluding participants who developed depression or dementia within the first year of follow-up (Supplementary Table S3), using different time intervals for participants entering different states on the same date (Supplementary Table S4), and additionally adjusting for the Townsend deprivation index (Supplementary Table S5).

## Discussion

In this large community-based longitudinal study from the UK Biobank, we found that (1) CR played a role in multiple disease transition stages, including from baseline to depression, depression to dementia or death, and baseline to death, especially among middle-aged participants, and (2) participants with high CR had longer depression-free years of life than those with low CR. To our knowledge, this is the first study that examined the influence of CR on the development of depression and temporal progression from depression to dementia and ultimately to death.

### Comparison with previous research and interpretation of our findings

Several cross-sectional studies using univariate analyses investigated the relationship between CR and depressive symptoms in older adults, showing that the level of CR (commonly indexed based on education, occupation, and cognitive activity) is negatively related to depressive symptom rating scale scores [28–30]. Furthermore, a few cohort studies linked high education, reduced time spent watching TV, or high engagement in social and leisure activities to lower depression risk [13, 31, 32]. However, these single components appear not enough to fully represent the CR construct influenced by a wide range of experiences in life [6], and the relationships between composite proxy measures of CR and incident depression and subsequent development of dementia have not been investigated yet.

In our study, we found that high CR was associated with about half the risk of depression in comparison with low CR. Further, high CR appeared to protect against the development of post-depression dementia. Nevertheless, this effect was attenuated and no longer statistically significant among older participants, consistent with our previous study reporting a risk of dementia buffered by a higher level of education in individuals with mid-life rather than late-life depression [4]. A possible explanation for the different pattern of results among middle-aged and older participants could be that depression occurring in older age is more likely to be part of the dementia prodrome [5, 33], and therefore the depression–dementia association in this context might not be affected by CR. In this study, our use of multistate model considering transitions of various disease stages and competing risks provides a deeper understanding of the role of CR in the dynamic course of depression development and the pattern of neuropsychiatric comorbidities.

We also observed that high CR was associated with a lower risk of mortality in participants without depression. Similarly, a previous report from the Rotterdam Study suggested that CR (incorporating multiple relevant factors) is negatively related to total mortality in community-dwelling older adults [34]. People with high CR tend to have higher socioeconomic status, and hence they might pay more attention to their health and have greater access to healthcare services, leading to a lower risk of death [23]. By contrast, there remains a lack of literature on such associations in people with depression. We found a significant association between CR and death in middle-aged participants with depression, but not older ones. Older adults with depression are reported to have a much higher risk of mortality than younger patients, possibly due to more severe vascular pathologies and structural brain abnormalities [8, 35, 36]. In this case, high CR might not be sufficient to buffer these detrimental impacts on health and survival.

Beyond morbidity, multimorbidity, and mortality, it is necessary to consider the influence of CR on quality of life using metrics such as disease-free survival. Such metrics could provide additional information when evaluating the overall health consequences of CR [37, 38]. Notably, our results showed that high CR was related to a 46% lower risk of incident depression or death and 2.77 years longer depression-free survival; that is to say, high CR might help maintain people in a relatively healthy status free of any progression to depression, post-depression dementia, or death. Also, these associations appeared more pronounced in middle-aged participants than older ones. Together with all the findings, our study underscores the contribution of CR to not only the primary prevention of depression but also the potential to mitigate the development of comorbidities like dementia and premature mortality after the diagnosis of depression, particularly at a younger age. This has important public health implications in light of the prevalent neuropsychiatric comorbidity with age.

The mechanisms underlying CR and its relation to depression and further to post-depression dementia remain poorly understood. One hypothesis is that the brain could use pre-existing cognitive processing approaches or recruit alternative neural regions and networks to compensate for brain pathology, which helps in maintaining psychological and cognitive functions [39]. In addition, evidence from animal models links environmental enrichment, defined as the generation of novelty and complexity in raising conditions that strengthen cognitive and sensory stimulation, to reduced intracerebral inhibition as well as increased expression and signaling of brain-derived neurotrophic factor [40–42]. These processes contribute to neural plasticity in the brain, which facilitates the defense against depression and brain aging [43, 44].

### Strengths and limitations

The main strength of the current study lies in the use of the multi-state model, yielding less biased estimates than the traditional Cox model, distinguishing the effect of CR on each stage in the progression trajectory of diseases, and assessing both etiological and prognostic factors simultaneously. Moreover, given that CR is a dynamic construct developing from diverse lifetime experiences that are not mutually exclusive but often interrelated to each other [6, 7], our use of a composite CR indicator captures the accumulation and interaction of different CR-related components. Limitations of this study also need to be considered. First, participants in the UK Biobank are generally highly educated and primarily white, so generalization to people from other socioeconomic or ethnic backgrounds should be undertaken with caution. Second, CR is a theoretical and hypothetical construct, and well-defined measures are not yet available. Additionally, there is no clear cutoff for the CR indicator, and CR categories might vary in different samples. Following previous studies [7, 15, 16], we used a latent variable approach based on real distributions of data and considered factors beyond educational level, the most straightforward and common proxy measure of CR. Third, we could not capture the changes in covariates and consider their effects on the observed associations in the modeling, because the information on covariates was collected at baseline and dealt with as time-fixed. Finally, incident depression and dementia cases were ascertained using register-based data, and thus some cases could not be captured. However, research has assessed the accuracy of using current resources to identify these diseases and has shown that these data are reliable enough for epidemiological studies [45, 46]. Besides, it could be challenging to precisely differentiate the onsets of depression and dementia, especially for older adults, because of their similar symptoms and delayed diagnoses. The temporality of the association between depression and dementia warrants further clarification, although the mean age difference between the two disorders was over three years.

## Conclusion

This study provides new evidence that a high level of CR is associated with lower risks of depression and subsequent dementia and death, especially in middle age. Higher CR may also prolong depression-free survival. Our findings underscore the importance of CR-promoting experiences and lifestyles for the multi-level prevention of depression and to support mentally healthier longevity.

## Supporting information

Yang et al. supplementary material Yang et al. supplementary material

## Data Availability

Access to UK Biobank data can be requested through a standard data access procedure. Requests to access these datasets should be directed to http://www.ukbiobank.ac.uk/register-apply.

## Supplementary material

The supplementary material for this article can be found at http://doi.org/10.1192/j.eurpsy.2024.1762.
