# Supplementary material for: Influence of cognitive reserve on risk of depression and subsequent dementia: A large community-based longitudinal study
Source: Eur Psychiatry. 2024 Jun 4;67(1):e45. doi: 10.1192/j.eurpsy.2024.1762 (PMC11441338; doi:10.1192/j.eurpsy.2024.1762)
Supplement: Yang et al. supplementary material [file S0924933824017620sup001.docx]

**Supplemental materials**

This Supplemental material was supporting the methods and results for the manuscript titled *Influence of cognitive reserve on risk of depression and subsequent dementia: A large community-based longitudinal study* by Yang et al.

**Supplementary Method 1** **Details on assessments of cognitive reserve-related variables and latent class analysis**

Educational level, occupational complexity, mentally passive sedentary behavior, social connection, confiding in others, and leisure activity engagement reflect different aspects of cognitive reserve (CR), and thus we used these six variables to generate an overall CR indicator. Relevant information was collected via computerized touch-screen questionnaires at baseline. If a category in the six variables occurred with a frequency of <10%, it would be merged with the closest category to yield a more informative one [1]. The answers “Prefer not to answer” and “Do not know” in these questions were set missing.

Educational level

Participants were asked to indicate whether they possessed one or more of the following qualifications: “college/university degree”, “A levels/AS levels or equivalent”, “O levels/GCSEs or equivalent”, “CSEs or equivalent”, “NVQ or HND or HNC or equivalent”, “Other professional qualifications eg: nursing, teaching”, and “None of the above”. We defined education level based on the maximum years of schooling corresponding to these qualifications [2], and classified it as 1) no educational qualifications, 2) Certificate of Secondary Education, O levels/General Certificate of Secondary Education, A levels/AS levels or equivalent, 3) other professional qualifications, 4) National Vocational Qualification, Higher National Diploma, Higher National Certificate or equivalent, or 5) college/university degree.

Occupational complexity

Participants were asked to indicate their job titles and employment status. Job titles were coded as 8-digit job codes and presented in a tree structure derived from the Standard Occupational Classification 2000 system, which were developed by the UK Office of National Statistics [3]. A truncated version of the fully detailed job codes was obtained where participants were grouped according to the first 4 digits of their job codes. The socio-economic classification (SEC) in analytic classes was directly derivable from these codes [4]; thus, SEC was an occupationally based classification. The simplified SEC was rated as the ordinal variables: 1.1 (large employers and higher managerial occupations), 1.2 (higher professional occupations), 2 (lower managerial and professional occupations), 3 (intermediate occupations), 4 (small employers and own account workers), 5 (lower supervisory and technical occupations), 6 (semi-routine occupations), 7 (routine occupations), and 8 (never worked and long-term unemployed), with lower score indicating higher occupational complexity and attainment [5]. Here, we invoked the simplified SEC to define occupational complexity. An additional category, reflecting the lowest level of occupational complexity, included those who were unemployed, looking after home and/or family, or unable to work because of sickness or disability. If participants only indicated that they were retired, their occupational complexity was treated as missing data. Occupational attainment was categorized as 1) never worked and long-term unemployed or SEC 7, 2) SEC 4-6, 3) SEC 3, 4) SEC 2, or 5) SEC 1.2 or SEC 1.1.

Mentally passive sedentary behavior

Participants were asked to indicate how many hours they spent watching television in a typical day. If the time participant spent on television viewing varied a lot, then the participant was asked to give the average time for a 24-hour day in the last 4 weeks. The following checks were performed: if <0 or >24 the answer was rejected; if >8 then the participant was asked to confirm the answer. Mentally passive sedentary behavior was defined by the time spent watching television and was quartiled as 1) ≥4, 2) 3-3.9, 3) 2-2.9, or 4) <2.

Social connection

Participants were required to indicate how often they made or received friend/family visits, with possible answers consisting of “almost daily”, “2-4 times a week”, “about once a week”, “about once a month”, “once every few months”, “never or almost never” or “no friends/family outside household”. Frequency of social connection was classified as 1) no friends/family outside household or about once a month or less, 2) about once a week, 3) 2-4 times a week, or 4) almost daily.

Confiding in others

Participants were asked to indicate how often they were able to confide in someone close to them, with possible answers consisting of “almost daily”, “2-4 times a week”, “about once a week”, “about once a month”, “once every few months”, or “never or almost never”. Frequency of confiding in others was defined as 1) never or almost never, 2) about once a month or less, 3) 1-4 times a week, or 4) almost daily.

Leisure activity engagement

Participants were asked to indicate which one or more from a list of activities they attended once a week or more often. These activities consisted of “sports club or gym”, “pub or social club”, “religious group”, “adult education class”, and “other group activity”. The level of leisure activity engagement was defined by total number of activities selected. Answers corresponding to “none of above” were coded as “0”. Leisure activity engagement was classified as three levels: 1) low (0), 2) moderate (1 activity), or 3) high (2–5 activities).

Latent class analysis (LCA) was conducted to create the CR indicator using the R (*poLCA* package). Likelihood ratio statistic *G*^2^, Akaike information criterion (AIC), and Bayesian information criterion (BIC) were used for model selection, with lower values indicating a more reasonable model. We conduct the models with one to six latent classes, and the statistic parameters are reported in the following table.

**Table 1 for supplementary method** ***G*^2^ statistics, AIC, and BIC in models with different numbers of latent classes**

| **Models** | ***G*^2^** | **AIC** | **BIC** |
| --- | --- | --- | --- |
| One-class model | 220642.90 | 7042279 | 7042488 |
| Two-class model | 58031.98 | 6879708 | 6880136 |
| Three-class model | 34670.55 | 6856387 | 6857035 |
| Four-class model | 24779.25 | 6846535 | 6847403 |
| Five-class model | 17845.46 | 6839641 | 6840729 |
| Six-class model | 15053.51 | 6836890 | 6838197 |

Furthermore, the mean posterior probabilities in models with three to six latent classes were examined to assess the uncertainty of posterior classification, with a value of 0.70 or more indicating an acceptable uncertainty. As shown in table below, all mean posterior probabilities from the three-latent-class model were all ≥0.70, and therefore the three-latent-class model was ideal for the uncertainty of posterior classification.

**Table 2 for supplementary method** **Mean posterior probabilities and class membership probabilities in models with three to six latent classes**

| **Probabilities** | **Latent class 1** | **Latent class 2** | **Latent class 3** | **Latent class 4** | **Latent class 5** | **Latent class 6** |
| --- | --- | --- | --- | --- | --- | --- |
| **Two-latent-class model** | | | | | | |
| Mean posterior probabilities | 0.88 | 0.90 | - | - | - | - |
| Class membership probabilities | 0.43 | 0.53 | - | - | - | - |
| **Three-latent-class model** | | | | | | |
| Mean posterior probabilities | 0.89 | 0.78 | 0.82 | - | - | - |
| Class membership probabilities | 0.36 | 0.42 | 0.22 | - | - | - |
| **Four-latent-class model** | | | | | | |
| Mean posterior probabilities | 0.80 | 0.68 | 0.74 | 0.72 | - | - |
| Class membership probabilities | 0.21 | 0.19 | 0.38 | 0.22 | - | - |
| **Five-latent-class model** | | | | | | |
| Mean posterior probabilities | 0.66 | 0.67 | 0.59 | 0.79 | 0.71 | - |
| Class membership probabilities | 0.20 | 0.27 | 0.13 | 0.20 | 0.20 | - |
| **Six-latent-class model** | | | | | | |
| Mean posterior probabilities | 0.60 | 0.60 | 0.65 | 0.77 | 0.57 | 0.64 |
| Class membership probabilities | 0.20 | 0.16 | 0.13 | 0.19 | 0.10 | 0.22 |

In the three-latent-class model, Latent class 3 had higher levels of education, occupational complexity, confiding in others, and leisure activity engagement as well as a lower level of mentally passive sedentary behavior; Latent class 2 had moderate levels of all CR-related factors. Additionally, there were overall less favorable levels of CR-related factors in Latent class 1. Accordingly, Latent class 3, Latent class 2, and Latent class 1 could be labelled “high CR”, “moderate CR”, and “low CR”, respectively (**Supplementary Table 1**).

References:

[1] Sinha P, Calfee CS, Delucchi KL. Practitioner's Guide to Latent Class Analysis: Methodological Considerations and Common Pitfalls. Crit Care Med 2021;49(1):e63-e79.

[2] Rietveld CA, Medland SE, Derringer J, et al. GWAS of 126,559 individuals identifies genetic variants associated with educational attainment. Science 2013;340(6139):1467-1471.

[3] Office for National Statistics. Standard Occupational Classification 2000, Volume 1: Structure and descriptions of unit groups. The Stationery Office; 2000.

[4] Office for National Statistics. The National Statistics Socio-economic Classification User Manual 2005. [https://www.ons.gov.uk/ons/guide-method/classifications/archived-standard-classifications/soc-and-sec-archive/the-national-statistics-socio-economic-classification%2D%2Duser-manual.pdf](%20https:/www.ons.gov.uk/ons/guide-method/classifications/archived-standard-classifications/soc-and-sec-archive/the-national-statistics-socio-economic-classification%2D%2Duser-manual.pdf). Accessed 10 Jul. 2023.

[5] Ko H, Kim S, Kim K, et al. Genome-wide association study of occupational attainment as a proxy for cognitive reserve. Brain 2022;145(4):1436-1448.

**Supplementary Table 1 Distribution characteristics of levels of cognitive reserve-related variables in three latent classes**

| **Characteristics** | **No. of subjects** | **Three-latent-class model** | | |
| --- | --- | --- | --- | --- |
|  |  | Latent class 1  (n=84983) | Latent class 2  (n=194912) | Latent class 3  (n=156337) |
| **Educational level** |  |  |  |  |
| No educational qualifications | 75966 (17.41) | 69022 (81.22) | 6944 (3.56) | 0 |
| CSEs, O levels/GCSE, A/AS levels or equivalent | 97624 (22.38) | 8412 (9.90) | 89212 (45.77) | 0 |
| Other professional qualifications | 52359 (12.00) | 912 (1.07) | 38255 (19.63) | 13192 (8.44) |
| NVQ, HND, HNC or equivalent | 67347 (15.44) | 5100 (6.00) | 60249 (30.91) | 1998 (1.28) |
| College/university degree | 142936 (32.77) | 1537 (1.81) | 252 (0.13) | 141147 (90.28) |
| **Occupational complexity** |  |  |  |  |
| Unemployed or routine occupations | 67561 (15.49) | 41111 (48.38) | 17645 (9.05) | 8805 (5.63) |
| Semi-routine occupations, small employers and own account workers, or lower supervisory and technical occupations | 82533 (18.92) | 27518 (32.38) | 44232 (22.69) | 10783 (6.90) |
| Intermediate occupations | 71738 (16.44) | 10323 (12.15) | 49538 (25.42) | 11877 (7.60) |
| Lower managerial and professional occupations | 117648 (26.97) | 4465 (5.25) | 54167 (27.79) | 59016 (37.75) |
| Higher managerial and professional occupations | 96752 (22.18) | 1566 (1.84) | 29330 (15.05) | 65856 (42.12) |
| **Mentally passive sedentary behavior (hours/day)** | | | | |
| ≥4 | 125681 (28.74) | 52443 (61.71) | 54378 (27.90) | 18560 (11.87) |
| 3-3.9 | 103193 (23.66) | 17000 (20.00) | 56248 (28.86) | 29945 (19.15) |
| 2-2.9 | 117399 (26.91) | 10258 (12.07) | 56617 (29.05) | 50524 (32.32) |
| <2 | 90259 (20.69) | 5282 (6.22) | 27669 (14.20) | 57308 (36.66) |
| **Social connection** |  |  |  |  |
| No or less than once a month | 95441 (21.88) | 16579 (19.51) | 36294 (18.62) | 42568 (27.23) |
| About once a week | 157051 (36.00) | 25208 (29.66) | 72488 (37.19) | 59355 (37.97) |
| 2-4 times a week | 133733 (30.66) | 26716 (31.44) | 64721 (33.21) | 42296 (27.05) |
| Almost daily | 50007 (11.46) | 16480 (19.39) | 21409 (10.98) | 12118 (7.75) |
| **Confiding in others** |  |  |  |  |
| Never or almost never | 64312 (14.74) | 20440 (24.05) | 26939 (13.82) | 16933 (10.83) |
| Less than about once a month | 46582 (10.68) | 8578 (10.09) | 21174 (10.86) | 16830 (10.77) |
| 1-4 times a week | 87362 (20.03) | 13875 (16.33) | 38192 (19.59) | 35295 (22.58) |
| Almost daily | 237976 (54.55) | 42090 (49.53) | 108607 (55.72) | 87279 (55.83) |
| **Leisure activity engagement** | | | | |
| Low | 130806 (29.99) | 34965 (41.14) | 57012 (29.25) | 38829 (24.84) |
| Moderate | 192316 (44.09) | 39804 (46.84) | 86810 (44.54) | 65702 (42.03) |
| High | 113110 (25.93) | 10214 (12.02) | 51090 (26.21) | 51806 (33.14) |
| **Categorization** |  | Low CR | Moderate CR | High CR |

Abbreviations: CSE, Certificate of Secondary Education; GCSE, General Certificate of Secondary Education; NVQ, National Vocational Qualification; HND, Higher National Diploma; HNC, Higher National Certificate; CR, cognitive reserve

**Supplementary Table 2** Numbers (percentages) of participants in transitions from baseline to depression, subsequently to dementia, and ultimately to death in relation to cognitive reserve level

| **Cognitive reserve** | **No. of cases (proportion)** **in transitions** | | | | |
| --- | --- | --- | --- | --- | --- |
|  | **Baseline to depression** | **Depression to dementia** | **Baseline to death** | **Depression to death** | **Post-depression dementia to death** |
| **All participants** | | | | | |
| Low (n=84983) | 4862 (5.72) | 246 (5.06) | 9214 (10.84) | 714 (14.69) | 96 (39.02) |
| Moderate (n=194912) | 7320 (3.76) | 235 (3.21) | 10613 (5.45) | 675 (9.22) | 85 (36.17) |
| High (n=156337) | 4378 (2.80) | 136 (3.11) | 6864 (4.39) | 337 (7.70) | 57 (41.91) |
| **Middle age (<60 years)** | | | | | |
| Low (n=30807) | 2243 (7.28) | 43 (1.92) | 1849 (6.00) | 226 (10.08) | 15 (34.88) |
| Moderate (n=112965) | 4566 (4.04) | 43 (0.94) | 3072 (2.72) | 261 (5.72) | 12 (27.91) |
| High (n=100596) | 2874 (2.86) | 24 (0.84) | 2362 (2.35) | 136 (4.73) | 8 (33.33) |
| **Older age (≥60 years)** | | | | | |
| Low (n=54176) | 2616 (4.83) | 203 (7.76) | 7365 (13.59) | 488 (18.65) | 81 (39.90) |
| Moderate (n=81947) | 2754 (3.36) | 192 (6.97) | 7541 (9.20) | 414 (15.03) | 73 (38.02) |
| High (n=55741) | 1504 (2.70) | 112 (7.45) | 4502 (8.08) | 201 (13.36) | 49 (43.75) |

**Supplementary Table 3** Hazard ratios (HRs) and 95% confidence intervals (CIs) for associations between cognitive reserve and transitions from baseline to depression, post-depression dementia, and death, after excluding participants who developed depression or dementia within the first year of follow-up

| **Cognitive reserve** | **HR (95% CI)** | | | | |
| --- | --- | --- | --- | --- | --- |
|  | **Baseline to depression** | **Depression to dementia** | **Baseline to death** | **Depression to death** | **Post-depression dementia to death** |
| Low | 1.00 | 1.00 | 1.00 | 1.00 | 1.00 |
| Moderate | 0.62 (0.60, 0.65) | 0.80 (0.61, 1.04) | 0.74 (0.70, 0.77) | 0.90 (0.82, 0.98) | 0.85 (0.68, 1.05) |
| High | 0.49 (0.47, 0.52) | 0.77 (0.62, 0.97) | 0.65 (0.62, 0.69) | 0.89 (0.80, 0.99) | 0.91 (0.70, 1.18) |

Models were adjusted for age, sex, race, smoking status, alcohol consumption, physical activity, body mass index, hypertension, diabetes, heart disease, and stroke.

**Supplementary Table 4** Hazard ratios (HRs) and 95% confidence intervals (CIs) for associations between cognitive reserve and transitions from baseline to depression, post-depression dementia, and death, using a 0.5 day of time intervals for participants entering different states on the same date

| **Cognitive reserve** | **HR (95% CI)** | | | | |
| --- | --- | --- | --- | --- | --- |
|  | **Baseline to depression** | **Depression to dementia** | **Baseline to death** | **Depression to death** | **Post-depression dementia to death** |
| Low | 1.00 | 1.00 | 1.00 | 1.00 | 1.00 |
| Moderate | 0.61 (0.59, 0.64) | 0.79 (0.61, 1.03) | 0.73 (0.70, 0.77) | 0.87 (0.79, 0.94) | 0.84 (0.68, 1.05) |
| High | 0.48 (0.46, 0.51) | 0.79 (0.63, 0.98) | 0.65 (0.62, 0.69) | 0.85 (0.76, 0.95) | 0.91 (0.71, 1.18) |

Models were adjusted for age, sex, race, smoking status, alcohol consumption, physical activity, body mass index, hypertension, diabetes, heart disease, and stroke.

**Supplementary Table 5** Hazard ratios (HRs) and 95% confidence intervals (CIs) for associations between cognitive reserve and transitions from baseline to depression, post-depression dementia, and death, further adjusting for the Townsend deprivation index

| **Cognitive reserve** | **HR (95% CI)** | | | | |
| --- | --- | --- | --- | --- | --- |
|  | **Baseline to depression** | **Depression to dementia** | **Baseline to death** | **Depression to death** | **Post-depression dementia to death** |
| Low | 1.00 | 1.00 | 1.00 | 1.00 | 1.00 |
| Moderate | 0.66 (0.64–0.69) | 0.85 (0.65–1.13) | 0.81 (0.77–0.86) | 0.89 (0.81–0.98) | 0.86 (0.70–1.08) |
| High | 0.53 (0.51–0.56) | 0.79 (0.62–0.98) | 0.78 (0.73–0.83) | 0.82 (0.73–0.92) | 0.97 (0.75–1.26) |

Models were adjusted for age, sex, race, smoking status, alcohol consumption, physical activity, body mass index, hypertension, diabetes, heart disease, and stroke.

502,412 participants aged 37-73 years

- 65,716 with a history of depression or dementia
- 268 developing depression after the occurrence of dementia
- 196 developing both dementia and depression on the same date

436,232 eligible for the current study

**Supplementary Figure 1** Flowchart of the study population
